# The FER rs4957796 TT genotype is associated with unfavorable 90-day survival in Caucasian patients with severe ARDS due to pneumonia

**DOI:** 10.1038/s41598-017-08540-7

**Published:** 2017-08-29

**Authors:** José Hinz, Benedikt Büttner, Fabian Kriesel, Maximilian Steinau, Aron Frederik Popov, Michael Ghadimi, Tim Beissbarth, Mladen Tzvetkov, Ingo Bergmann, Ashham Mansur

**Affiliations:** 1 Department of Anaesthesiology, University Medical Centre, Georg August University, Robert-Koch-Str.40, D-37075 Goettingen, Germany; 2 Department of Cardiothoracic Transplantation & Mechanical Support, Royal Brompton and Harefield Hospital, Harefield, Hill End Road, UB9 6JH London, United Kingdom; 3 Department of Cardiothoracic and Vascular Surgery, University of Frankfurt, Theodor-Stern-Kai 7, D-60590 Frankfurt, Germany; 4 Department of General and Visceral Surgery, University Medical Centre, Georg August University, D-37075 Goettingen, Germany; 5 Department of Medical Statistics, University Medical Centre, Georg August University, Robert-Koch-Str.40, D-37075 Goettingen, Germany; 6 Institute of Clinical Pharmacology, University Medical Center, Georg August University, Goettingen, Germany

## Abstract

A recent genome-wide association study showed that a genetic variant within the FER gene is associated with survival in patients with sepsis due to pneumonia. Because severe pneumonia is the main cause of acute respiratory distress syndrome (ARDS), we aimed to investigate the effect of the FER polymorphism rs4957796 on the 90-day survival in patients with ARDS due to pneumonia. An assessment of a prospectively collected cohort of 441 patients with ARDS admitted to three intensive care units at the University Medical Centre identified 274 patients with ARDS due to pneumonia. The 90-day mortality risk was recorded as the primary outcome parameter. Sepsis-related organ failure assessment (SOFA) scores and organ support-free days were used as the secondary variables. FER rs4957796 TT-homozygous patients were compared with C-allele carriers. The survival analysis revealed a higher 90-day mortality risk among T homozygotes than among C-allele carriers (p = 0.0144) exclusively in patients with severe ARDS due to pneumonia. The FER rs4957796 TT genotype remained a significant covariate for the 90-day mortality risk in the multivariate analysis (hazard ratio, 4.62; 95% CI, 1.58–13.50; p = 0.0050). In conclusion, FER rs4957796 might act as a prognostic variable for survival in patients with severe ARDS due to pneumonia.

## Introduction

Acute respiratory distress syndrome (ARDS) is characterized by excessive and protracted pulmonary inflammation with increased permeability of pulmonary capillary and alveolar epithelial cells, leading to hypoxemia that is refractory to the usual oxygen therapy^1, 2^. ARDS represents a common clinical problem in ICU patients and is associated with a short-term mortality of up to 45%^3, 4^ and significant long-term morbidity^5^.

Attempts to reduce the mortality in ARDS patients by decreasing the overwhelming pulmonary inflammation have proven mostly disappointing^6–8^, most likely because these interventions have usually been applied unselectively to heterogeneous groups of patients, without considering the potential influence of host genetic diversity on the response to treatment. Genomics has the potential to substantially advance our understanding of the key biological pathways implicated in human disease and to suggest new targets for treatment or prevention^9^. Additionally, the characterization of genetic variants associated with the outcome of sepsis could enable identification of those at high risk who might benefit from more aggressive interventions or from specific, individually targeted, early, or pre-emptive measures.

A recent genome-wide association study has shown that a genetic variant within the intronic region of the FER gene, rs4957796, is associated with survival in patients with sepsis due to pneumonia. The FER gene encodes a non-receptor protein tyrosine kinase that acts downstream of cell-surface receptors for growth factors and is ubiquitously expressed^10^. FER is known to play a role in the regulation of the actin cytoskeleton, cell adhesion, migration and invasion, and chemotaxis^11–14^. FER impacts leucocyte recruitment and intestinal barrier dysfunction in response to bacterial lipopolysaccharides^15, 16^, findings relevant to the potential mechanisms through which variants in this gene could influence sepsis survival. Furthermore, studies in mice targeted with an FER kinase-inactivating mutation have shown that FER can inhibit neutrophil chemotaxis^17^. Neutrophil recruitment to the site of infection is essential in innate immune defense, and changes in relevant signaling pathways could lead to a failure to clear bacterial infections or the promotion of further tissue damage^18^.

Because the most frequent lung condition leading to ARDS is sepsis due to pneumonia^19, 20^, this study aimed to investigate the effect of the FER rs4957796 variant on the 90-day survival in patients with ARDS due to pneumonia according to the severity of ARDS.

## Results

### Baseline characteristics

A total of 274 adult Caucasian patients with ARDS due to pneumonia were enrolled in this study. The patients’ ages ranged from 18 to 90 years (median, 62 years) (Table 1). Thirty-one percent of the patients were women, and 69% were men. The genotype distribution of FER rs4957796 was 10:79:185 (CC:CT:TT), which is consistent with Hardy-Weinberg equilibrium (p = 0.9111). The minor allele frequency was 18%. The FER rs4957796 CC and CT genotypes were pooled to explore the clinical effect of the TT genotype compared to that of C-allele carriers in accordance with our a priori hypothesis (Table 1). The distribution values of sepsis/severe sepsis and septic shock were 21% and 79%, respectively. At baseline, the mean SOFA and APACHE II morbidity scores were 9.4 ± 3.3 and 21.8 ± 6.3, respectively (Table 1). The frequencies of organ support therapies including mechanical ventilation, vasopressor therapy and renal replacement therapy at sepsis onset were 93%, 65% and 7%, respectively (Table 1).

**Table 1:** Patients’ baseline characteristics.

Parameter	All (n = 274)	ARDS (all patients)		p value
		T/C+C/C	T/T	
		(n=89)	(n=185)	
Age, years	61 ± 15	62 ± 16	60 ± 15	0.0967
Male [%]	69	66	70	0.5638
Body mass index	28 ± 7	27 ± 5	28 ± 8	0.6523
**Severity of sepsis**				
Sepsis/severe sepsis, %	21	27	18	0.1032
Septic shock, %	79	73	82	0.1032
Sequential Organ Failure Assessment (SOFA) score	9.4 ± 3.3	9.5 ± 3.1	9.3 ± 3.4	0.7312
Acute Physiology and Chronic Health Evaluation (APACHE II) score	21.8 ± 6.3	22.0 ± 6.2	21.6 ± 6.3	0.6409
**Comorbidities [%]**				
Hypertension	54	57	53	0.5003
History of myocardial infarction	7	7	6	0.9356
Chronic obstructive pulmonary disease	18	22	15	0.1346
Renal dysfunction	9	10	9	0.8071
Noninsulin-dependent diabetes mellitus	9	8	10	0.6157
Insulin-dependent diabetes mellitus	9	8	10	0.6157
Chronic liver disease	4	2	4	0.3905
History of cancer	15	15	15	0.9086
History of stroke	6	9	4	0.1231
**Recent surgical history [%]**				0.9169
Elective surgery	25	24	25	
Emergency surgery	54	56	54	
No history of surgery	21	20	21	
**Organ support [%]**				
Used during observation period				
Mechanical ventilation	99	100	99	0.3249
Use of vasopressor	79	73	82	0.1032
Renal replacement therapy	19	17	20	0.5340
Used on sepsis onset				
Mechanical ventilation	93	96	92	0.2702
Use of vasopressor	65	63	66	0.5615
Renal replacement therapy	7	7	7	0.9306
Use of statins	29	38	24	0.0176

### Outcomes

#### Mortality

**Figure 1:**
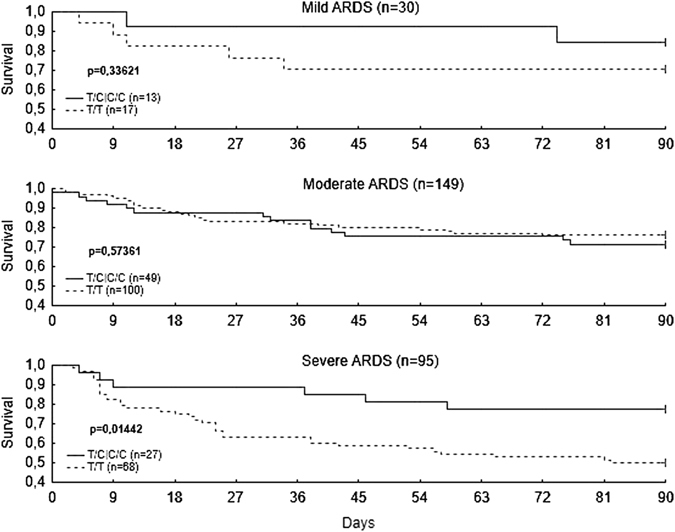
Kaplan-Meier survival analysis. Kaplan-Meier survival analysis of 90-day survival according to FER rs4957796 for the three acute respiratory distress syndrome (ARDS) groups. FER polymorphism rs4957796 is associated with higher 90-day mortality exclusively in patients with severe ARDS due to pneumonia (p = 0.0144, log-rank test).

To detect the effect of FER rs4957796 on the 90-day outcome of ARDS and the dependence on ARDS severity (mild, moderate, and severe), Kaplan-Meier survival analysis was performed for the 90-day survival in these three groups of patients (Fig. 1); FER rs4957796 affected the 90-day survival exclusively among patients with severe ARDS; patients with severe ARDS who were C-allele carriers showed a lower 90-day mortality rate than TT-homozygous patients (p = 0.0144) (Fig. 1).

### Multivariate analysis

To exclude the effects of potential confounders and different baseline variables on the 90-day mortality among patients with severe ARDS, the baseline patient characteristics in the severe ARDS group were analyzed according to the FER rs4957796 genotype (Table 2). A multivariate Cox regression model including different baseline variables and relevant confounders revealed the highest hazard ratio for patients with the FER rs4957796 TT genotype (hazard ratio, 4.62; 95% CI, 1.58–13.50; p = 0.0050) (Table 3), followed by lack of statin use (hazard ratio, 2.31; 95% CI, 0.85–6.30; p = 0.1024) and a poor SOFA score (hazard ratio, 1.09; 95% CI, 0.96–1.24; p = 0.1784) (Table 3). This finding indicates that despite potential baseline confounders (age, gender, initial APACHE II and SOFA scores, and history of stroke and statin therapy), the FER rs4957796 TT genotype is an independent prognostic variable for outcome and exhibits the most significant effect on the 90-day mortality (Table 3).

**Table 2:** Severe acute respiratory distress syndrome patients’ baseline characteristics.

Parameter	All (n = 95)	Severe ARDS		p value
		T/C+C/C	T/T	
		(n = 27)	(n = 68)	
Age, years	58 ± 15	59 ± 15	58 ± 15	0.6430
Male [%]	68	70	68	0.7967
Body mass index	29 ± 8	27 ± 4	30 ± 9	0.5966
**Severity of sepsis**				
Sepsis/severe sepsis, %	9	19	6	0.0578
Septic shock, %	91	81	94	
Sequential Organ Failure Assessment (SOFA) score	23.5 ± 6.2	23.4 ± 7.1	23.6 ± 5.9	0.8794
Acute Physiology and Chronic Health Evaluation (APACHE II) score	10.8 ± 3.2	10.6 ± 3	10.9 ± 3.3	0.7336
**Comorbidities [%]**				
Hypertension	56	52	57	0.6262
History of myocardial infarction	3	4	3	0.8479
Chronic obstructive pulmonary disease	18	15	19	0.6216
Renal dysfunction	6	11	4	0.2259
Noninsulin-dependent diabetes mellitus	9	7	10	0.6647
Insulin-dependent diabetes mellitus	8	7	9	0.8226
Chronic liver disease	2	4	1	0.4940
History of cancer	20	22	19	0.7329
History of stroke	7	15	4	0.0800
**Recent surgical history [%]**				0.1643
Elective surgery	25	37	21	
Emergency surgery	45	44	46	
No history of surgery	29	19	34	
**Organ support [%]**				
Used during observation period				
Mechanical ventilation	100	100	100	
Use of vasopressor	91	81	94	0.0578
Renal replacement therapy	29	26	31	0.6327
Used on sepsis onset				
Mechanical ventilation	98	96	99	0.4941
Use of vasopressor	77	74	78	0.6869
Renal replacement therapy	7	11	6	0.3789
Use of statins	23	37	18	0.0433

**Table 3:** Cox regression analysis of severe acute respiratory distress syndrome patients.

Variable	Hazard ratio	95% CI	p value
Age	1.00	0.98–1.02	0.9720
Male gender	1.01	0.49–2.07	0.9724
Body mass index	0.98	0.94–1.02	0.3415
Sequential Organ Failure Assessment (SOFA) score	1.09	0.96–1.24	0.1784
Acute Physiology and Chronic Health Evaluation (APACHE II) score	1.05	0.99–1.12	0.0873
History of stroke	0.41	0.09–1.86	0.2500
No statin therapy	2.31	0.85–6.30	0.1024
T/T genotype	4.62	1.58–13.50	0.0050

### Disease severity

To assess the effect of FER rs4957796 on disease severity and organ dysfunction over the first 28 days in the ICU, organ-specific SOFA score analysis was conducted; no differences were found in the organ-specific SOFA scores between the genotypes of FER rs4957796 (Table 4). Similarly, the requirement of organ support as measured by organ support-free days did not differ between the two FER rs4957796 groups. Adjustment for potential confounder and different baseline characteristic revealed no significant differences in organ-specific SOFA scores and the need of organ support (Supplementary information).

**Table 4:** Disease severity among patients with severe acute respiratory distress syndrome.

Variable	All	Severe ARDS		p Value
	(n = 95)	T/C|C/C	T/T	
		(n = 27)	(n = 68)	
SOFA	8.6 ± 3.7	7.8 ± 2.9	8.9 ± 3.9	0.3102
SOFA-Respiratory score	2.6 ± 0.5	2.5 ± 0.6	2.6 ± 0.5	0.1682
SOFA-Cardiovascular score	1.8 ± 1	1.7 ± 0.9	1.9 ± 1.1	0.8141
SOFA-Central nervous system score	2.4 ± 1	2.1 ± 1	2.5 ± 0.9	0.0571
SOFA-Renal score	0.8 ± 1	0.8 ± 0.8	0.8 ± 1.1	0.6986
SOFA-Coagulation score	0.4 ± 0.6	0.2 ± 0.4	0.5 ± 0.7	0.1340
SOFA-Hepatic score	0.3 ± 0.7	0.4 ± 0.6	0.3 ± 0.7	0.7590
Length of stay in ICU, days	21 ± 16	19 ± 12	22 ± 18	0.4523
**Organ support-free days**				
Ventilator-free days	2 ± 3	3 ± 2	2 ± 3	0.0628
Dialysis-free days	15 ± 8	15 ± 7	16 ± 8	0.6981
Vasopressor-free days	10 ± 7	10 ± 5	10 ± 7	0.8851
ECMO-free days	16 ± 9	16 ± 8	15 ± 9	0.5541
**Inflammatory values**				
Leucocytes (1000/µl)	13 ± 4	12 ± 3	13 ± 4	0.2853
CRP (mg/l)	164 ± 107 (35)	113 ± 86 (8)	179 ± 110 (27)	0.1882
Procalcitonin (ng/dl)	4.4 ± 10.9 (90)	3.2 ± 7.6 (24)	4.9 ± 11.9 (66)	0.4434
**Kidney values**				
Urine output (ml/day)	2955 ± 1264	3388 ± 1267	2783 ± 1231	0.1342
Urine output (ml/kg/h)	1.4 ± 0.7	1.7 ± 0.7	1.3 ± 0.6	0.0165
Creatinine (mg/dl)	1.2 ± 0.7	1.3 ± 0.7	1.2 ± 0.7	0.4552
**Liver values**				
AST (GOT) (IU/l)	426 ± 1430 (60)	296 ± 588 (14)	465 ± 1605 (46)	0.2451
ALT (GPT) (IU/l)	124 ± 243 (93)	157 ± 329	111 ± 199 (66)	0.8689
Bilirubin (mg/dl)	1.4 ± 2.9	1.1 ± 1.4	1.6 ± 3.3	0.7917

## Discussion

This observational study addresses the question of whether the FER polymorphism rs4957796 is associated with 90-day survival in patients with sepsis-associated ARDS due to pneumonia and its association with the severity of ARDS (mild, moderate, and severe). The main finding of this investigation was that FER rs4957796 TT-homozygous patients with severe ARDS have a significantly higher mortality risk than C-allele carriers.

Our finding of a deleterious effect of the FER rs4957796 TT genotype on survival in patients with severe ARDS due to pneumonia is consistent with the results of a recent GWA showing that the T-allele of FER rs4957796 was associated with increased mortality in patients with sepsis due to pneumonia in four independent European cohorts^21^.

FER rs4957796 polymorphism is a non-coding variant localized in intron 19 of the gene. Until now there are no records regarding this polymorphism in the eQTL data bases (http://genenetwork.nl/bloodeqtlbrowser/, status 16–6–2017) or in ExSNP (http://www.exsnp.org/ status 16–6–2017). In addition, there are no indications for special regulatory features in close proximity based on DNase hypersensitivity or specific histone acetylation patterns (Supplementary Fig. 1). However, rs4957796 is in a linkage disequilibrium with variants spanning several coding regions^22^ and thus may be only a marker for a linked functional variant. Our study confirms the association of rs4957796 polymorphism with sepsis and underlines the necessity of functional analyses to reveal the biological mechanism behind these associations. According to a recent study by Dolgachev, *et al*.^23^ showing that FER gene delivery affects survival in a mouse model of combined lung contusion and pneumonia and improves the efficiency of bacterial clearance within contused lungs, we hypothesize that the T-allele might be associated with lower FER gene expression, causing the observed poor outcome among ARDS patients.

The observed effect of FER rs4957796 on the 90-day survival exclusively in patients with severe sepsis-associated ARDS due to pneumonia is consistent with several investigations showing that ARDS subgroups (mild, moderate, and severe) are associated with clearly definable distinct histopathological features that may affect the clinical course and outcome of ARDS patients^24^. Studies aiming to investigate the effects of patient characteristics on the outcome of ARDS should follow a differentiated approach to assess the three ARDS subgroups separately, e.g., according to our own prospective investigation, statin therapy affects mortality nearly exclusively in patients with severe ARDS, with no significant effect in either the mild or moderate ARDS groups^25^.

Because the clinical course and mortality in patients with sepsis and ARDS are affected by several factors, e.g., comorbidities^26, 27^, comedications, and treatment modalities, the detected independent strong effect of the rs4957796 TT genotype on mortality risk indicates that this polymorphism should be considered in the clinical routine to identify patients at risk and in future studies investigating the outcome of patients with sepsis and ARDS caused by pneumonia.

The lack of an effect of the unfavorable genotype rs4957796 TT on disease severity (secondary variables) within 28 days in the ICU, as measured by SOFA sub-score analysis and organ support-free days, may be due to the possibility that the FER protein may exert deleterious effects on the lungs of ARDS patients and on other organ systems that cannot yet be detected using standard clinical assessment tools, e.g., SOFA pulmonary score or ventilator-free days.

This study has some limitations. Because the effect of the polymorphism on the 90-day mortality in patients with sepsis-associated ARDS due to pneumonia according to disease severity was unknown, we could not conduct power calculations at the beginning of the investigation to determine a sample size with adequate power. However, an ad hoc power analysis revealed a power of 0.83 in the severe ARDS group, considering the observed mortalities of 22% and 50% among the TT-homozygous and C-allele carriers, respectively. Thus, our sample size was sufficiently large to adequately address the objective. Another potential limitation is that this study was conducted in a single center. Thus, our findings must be further validated in independent cohorts from other centers to assess their generalizability. Although one of the major strengths of our investigation is that we have assessed a well-defined homogeneous cohort of patients with sepsis-associated ARDS caused by pneumonia, it has become evident that assessing such homogeneous cohorts from a single center is particularly advantageous because it is the best way to control confounding evoked by potential inter-center heterogeneity.

To the best of our knowledge, this is the first investigation to assess the effect of FER rs4957796 on mortality among patients with sepsis-associated ARDS caused by pneumonia. The observed independent increased risk for the 90-day mortality in patients with severe ARDS may help identify patients at risk in clinical settings.

## Methods

### Patients

The patients were recruited through the GENOSEP database of the Department of Anaesthesiology at the University Medical Centre, Goettingen, Germany. This database comprises a prospectively collected cohort of patients with sepsis. As described previously, consecutive adult patients admitted to the three surgical ICUs of the University Medical Centre between April 2012 and May 2015 were screened daily according to the American College of Chest Physicians/Society of Critical Care Medicine (ACCP/SCCM) criteria for sepsis, severe sepsis, or septic shock^28, 29^. Patients with sepsis were screened daily according to the Berlin definition of ARDS to identify those with sepsis-associated ARDS^24, 25, 28^. According to the PaO2/FiO2 ratio, patients with ARDS were classified into three categories: mild ARDS, 201–300 mmHg (≤39.9 kPa); moderate ARDS, 101–200 mmHg (≤26.6 kPa); and severe ARDS, ≤ 100 mmHg (≤13.3 kPa). Finally, patients with sepsis-associated ARDS caused by pneumonia were included in this study.

As described previously, the exclusion criteria were as follows: (1) age younger than 18 years; (2) pregnancy or breastfeeding; (3) immunosuppressive therapy; (4) acute myocardial infarction within the past 6 weeks; (5) congestive heart failure, classified as New York Heart Association functional class IV; (6) HIV infection; (7) a do not resuscitate or do not treat order; (8) very likely to die within 28 days due to end-stage uncorrectable disease; (9) a persistent vegetative state; (10) participation in an interventional trial; and (11) study-site employee or a family member of a study-site employee. This investigation was approved by the institutional ethics committee of the University of Goettingen in Goettingen, Germany, and was performed in accordance with the provisions of the Declaration of Helsinki. The study was performed in accordance with relevant guidelines and regulations. The methods were carried out in accordance with the approved guidelines. Written informed consent was obtained either from the patient or their legal representative.

### Data collection and clinical endpoints

The baseline characteristics of the patients were recorded upon enrolment and included all relevant comorbid conditions, type of sepsis, recent surgical history, and organ support. The patients were followed up for 90 days, and the mortality risk within this period was assessed as the primary outcome variable. The Sequential Organ Failure Assessment (SOFA)^30^ and Acute Physiology and Chronic Health Evaluation (APACHE) II^31^ scores were evaluated at sepsis onset. Morbidity was assessed over 28 days in the ICU via recording of the organ-specific SOFA scores and the need for organ support (mechanical ventilation, vasopressor therapy, and renal replacement therapy) as the secondary outcome variable. Given that the majority of patients leave the ICU within 28 days (survive or decease), our study focused on the clinical progression within the first 28 days in the ICU. Clinical data were obtained from the electronic patient record system (IntelliSpace Critical Care and Anaesthesia (ICCA); Philips Healthcare, Andover, MA, USA).

### FER rs4957796 genotyping

DNA was extracted by automated solid-phase extraction from 350 µl of EDTA whole blood using an EZ1® DNA Blood Kit in BioRobot EZ1® or from PBMCs using an AllPrep DNA Mini Kit according to the manufacturer’s instructions (all from Qiagen, Hilden, Germany). The DNA quantity and quality were determined spectrophotometrically. Genotyping was performed using the pre-designed TaqMan® SNP genotyping assay C__28002866_10 according to the manufacturer’s instructions (Life Technology, Darmstadt, Germany).

A total of 15% of the samples were genotyped in duplicate, yielding results that showed complete concordance. The observed genotypes were in Hardy-Weinberg equilibrium. The identity of the DNA samples was controlled by sex-typing and showed 100% concordance between the initially documented and the genetically determined sex^32^.

### Statistical analyses

Statistical analyses were performed using Statistica software (version 12; StatSoft, Tulsa, Oklahoma, USA). The significance of categorical variables was calculated using two-sided Fisher’s exact or chi-squared tests, as appropriate. Two continuous variables were compared using the Mann-Whitney test. Time-to-event data were compared using the log-rank test from the Statistica package for Kaplan-Meier survival analysis. To exclude the effects of potential confounders (age, gender and body mass index (BMI), and morbidity scores (SOFA and APACHE II), statin therapy^25^ and covariates that varied at baseline (differently distributed comorbidities with p-values < 0.1) on survival, we performed a multivariate Cox regression analysis to examine the survival time. A power calculation was conducted using the Statistica package for power analysis. A p value < 0.05 was considered statistically significant

### Data availability

The datasets generated during and/or analyzed during the current study are available from the corresponding author on reasonable request.

## Electronic supplementary material

Supplementary information
