# Supplementary material for: The FER rs4957796 TT genotype is associated with unfavorable 90-day survival in Caucasian patients with severe ARDS due to pneumonia
Source: Sci Rep. 2017 Aug 29;7:9887. doi: 10.1038/s41598-017-08540-7 (PMC5575093; doi:10.1038/s41598-017-08540-7)
Supplement: Supplementary file 1 — Supplementary information [file 41598_2017_8540_MOESM1_ESM.pdf]

Supplementary information

The FER rs4957796 TT genotype is associated with unfavorable 90-day survival in Caucasian patients with severe ARDS due to pneumonia

José Hinz, MD, PhD[1$], Benedikt Büttner, MD[1$], Fabian Kriesel, MD[1], Maximilian Steinau, MD[1], Aron Frederik Popov, MD, PhD, [2,3], Michael Ghadimi, MD, PhD[4], Tim Beissbarth, PhD[5], Mladen Tzvetkov, PhD[6], Ingo Bergmann, MD, PhD[1$], and Ashham Mansur, MD, PhD[*1$]

[1]Department of Anaesthesiology, University Medical Centre, Georg August University, Robert-Koch-Str.40, D-37075 Goettingen, Germany

[2]Department of Cardiothoracic Transplantation & Mechanical Support, Royal Brompton and Harefield Hospital, Harefield, Hill End Road, UB9 6JH London, United Kingdom

[3]Department of Cardiothoracic and Vascular Surgery, University of Frankfurt, Theodor-Stern-Kai 7, D-60590 Frankfurt, Germany

[4]Department of General and Visceral Surgery, University Medical Centre, Georg August University, D-37075 Goettingen, Germany

[5]Department of Medical Statistics, University Medical Centre, Georg August University, Robert-Koch-Str.40, D-37075 Goettingen, Germany

[6]Institute of Clinical Pharmacology, University Medical Center, Georg August University, Goettingen, Germany

[$]Equal contribution

1. Baseline Characteristica – mild ARDS

Parameter	All (n=30)	Severe ARDS T/C+C/C (n=13)	T/T (n=17)	p value
Age, years	61±15	61±17	61±14	1.0000
Male [%]	77	77	76	0.9768
Body Mass Index	26±5	27±7	26±3	0.4898
**Severity of sepsis**				
Sepsis/severe sepsis, %	37	38	35	0.8584
Septic shock, %	63	62	65	
Sequential Organ Failure Assessment score	6.6±2.8	6.8±2.6	6.5±3.1	0.9068
Acute Physiology and Chronic Health Evaluation (APACHE II) score	17.4±5.5	17.7±5.6	17.1±5.6	0.8667
**Comorbidities [%]**				
Hypertension	43	38	47	0.6377
History of myocardial infarction	3	0	6	0.3738
Chronic obstructive pulmonary disease	23	23	24	0.9768
Renal dysfunction	13	15	12	0.7726
Noninsulin-dependent diabetes mellitus	0	0	0	1.0000
Insulin-dependent diabetes mellitus	0	0	0	1.0000
Chronic liver disease	3	0	6	0.3738
History of cancer	13	23	6	0.1700
History of stroke	0	0	0	1.0000
**Recent surgical history[%]**				0.9741
Elective surgery	30	30	29	
Emergency Surgery	63	62	65	
No History of Surgery	7	8	6	
**Organ support [%]**				
Used during observation period				
Mechanical ventilation	97	100	94	0.3738
Use of vasopressor	63	62	65	0.8584
Renal replacement therapy	0	0	0	1.0000
Used on sepsis onset				
Mechanical ventilation	83	100	70	0.0322
Use of vasopressor	0	0	0	1.0000
Renal replacement therapy	47	38	53	0.4308
Use of Statins[%]	27	23	29	0.6974

2. Baseline Characteristica – moderate ARDS

Parameter	All (n=149)	Severe ARDS T/C+C/C (n=49)	T/T (n=100)	p value
Age, years	62±16	64±17	61±15	0.0783
Male [%]	67	61	70	0.2841
Body Mass Index	27±7	27±4	28±8	0.6776
**Severity of sepsis**				
Sepsis/severe sepsis, %	25	29	24	0.5476
Septic shock, %	75	71	76	
Sequential Organ Failure Assessment score	9.3±3.4	9.7±3.6	9.1±3.3	0.4186
Acute Physiology and Chronic Health Evaluation (APACHE II) score	21.5±6.0	22.4±5.4	21.0±6.2	0.2504
**Comorbidities [%]**				
Hypertension	56	65	51	0.0986
History of myocardial infarction	9	10	9	0.8129
Renal dysfunction	11	8	12	0.4773
Noninsulin-dependent diabetes mellitus	11	10	12	0.7460
Insulin-dependent diabetes mellitus	11	10	11	0.8828
Chronic liver disease	5	2	6	0.2833
History of cancer	12	8	14	0.3044
History of stroke	6	8	5	0.4464
**Recent surgical history[%]**				0.1579
Elective surgery	23	14	27	
Emergency Surgery	58	61	57	
No History of Surgery	19	25	16	
**Organ support [%]**				
Used during observation period				
Mechanical ventilation	99	100	99	0.4825
Use of vasopressor	75	71	76	0.5476
Renal replacement therapy	16	16	16	0.9594
Used on sepsis onset				
Mechanical ventilation	92	94	91	0.5443
Use of vasopressor	62	63	61	0.7892
Renal replacement therapy	8	6	9	0.5443
Use of Statins[%]	33	43	28	0.0698

3. Cox Regression – mild ARDS*

Variable	Hazard ratio	95 % CI	*p* value
Age	1.20	0.98-1.47	0.0774
Male gender	30.58	0.03-35797.31	0.3427
Body mass index	0.59	0.24-1.48	0.2636
Sequential Organ Failure Assessment	0.43	0.15-1.27	0.1263
Acute Physiology and Chronic Health Evaluation (APACHE II)	1.18	0.81-1.75	0.3756
No statin therapy	0.00	0.00-24.00	0.2083
T/T Genotype	1.65	0.06-45.82	0.7690

*To exclude the effects of potential confounders (age, gender and body mass index (BMI), and morbidity scores (SOFA and APACHE II), statin therapy[25] and covariates that varied at baseline (differently distributed comorbidities with p-values < 0.1) on survival, we performed a multivariate Cox regression analysis to examine the survival time

4. Cox Regression – moderat ARDS*

Variable	Hazard ratio	95 % CI	*p* value
Age	1.09	1.05-1.14	0.0001
Male gender	0.42	0.17-1.02	0.0549
Body mass index	1.00	0.95-1.07	0.8826
Sequential Organ Failure Assessment	1.16	1.05-1.28	0.0023
Acute Physiology and Chronic Health Evaluation (APACHE II)	1.02	0.96-1.09	0.4483
Hypertension	1.54	0.67-3.50	0.3073
No statin therapy	1.00	0.46-2.19	0.9984
T/T Genotype	0.64	0.30-1.40	0.2686

*To exclude the effects of potential confounders (age, gender and body mass index (BMI), and morbidity scores (SOFA and APACHE II), statin therapy[25] and covariates that varied at baseline (differently distributed comorbidities with p-values < 0.1) on survival, we performed a multivariate Cox regression analysis to examine the survival time

5. Disease severity – mild ARDS (Adjusted)

Linear regression – with Age, sex, BMI, SOFA_d1, Apache, Statin and rs4957796 genotype.

Variable	All	mild ARDS		p Value
		T/C|C/C	T/T	
	(n=30)	(n=13)	(n=17)	
SOFA	4.5±1.6	4.6±1.6	4.5±1.7	0.5388
SOFA-Respiratory score	1.4±0.5	1.4±0.5	1.4±0.4	0.7511
SOFA-Cardiovascular score	1.1±0.7	1.1±0.6	1.1±0.8	0.8524
SOFA-Central nervous system score	1.6±1.2	1.6±1.1	1.6±1.3	0.7037
SOFA-Renal score	0.3±0.6	0.3±0.5	0.3±0.6	0.9530
SOFA-Coagulation score	0.1±0.3	0.1±0.3	0.1±0.3	0.8360
SOFA-Hepatic score	0.1±0.4	0.1±0.4	0.1±0.4	0.7274
Length of stay in ICU days	12±10	12±8	12±11	0.8904
28-day mortality (%)	17	8	24	0.2228
**Organ support-free days**				
Ventilator-free days	4±5	4±5	5±5	0.6643
Dialysis-free days	11±7	12±8	11±7	0.8904
Vasopressor-free days	10±7	10±7	9±7	0.8871
ECMO-free days	11±7	12±8	11±7	0.8904
**Inflammatory values**				
Leucocytes (1000/µl)	12±5	11±4	13±6	0.5651
CRP (mg/l)	162±126 (9)	208±192 (3)	139±93 (6)	n.a.
Procalcitonin (ng/dl)	1.6±5.1 (27)	3.3±8.2 (10)	0.7±1.0	0.1612
**Kidney values**				
Urine output (ml/day)	3055±729	2928±788	3153±688	0.2469
Urine output (ml/kg/hr)	1.6±0.5	1.5±0.5	1.7±0.4	0.5077
Creatinine (mg/dl)	0.9±0.5	0.9±0.5	0.9±0.6	0.9903
**Liver values**				
AST (GOT) (IU/l)	85±114 (10)	43±30 (4)	112±144 (6)	0.1986
ALT (GPT) (IU/l)	79±101 (29)	58±36	96±132 (16)	0.2145
Bilirubin (mg/dl)	0.7±0.5	0.6±0.4	0.7±0.5	0.5254

6. Disease severity – moderate ARDS (Adjusted)

Linear regression – with Age, sex, BMI, SOFA_d1, Apache, Statin , Hypertension (varied comorbidity at baseline) and rs4957796 genotype.

Variable	All	mild ARDS		p Value
		T/C|C/C	T/T	
	(n=149)	(n=49)	(n=100)	
SOFA	6.5±2.6	6.7±2.3	6.4±2.7	0.9100
SOFA-Respiratory score	2.0±0.6	2.0±0.6	1.9±0.6	0.6267
SOFA-Cardiovascular score	1.3±0.8	1.3±0.7	1.3±0.9	0.4835
SOFA-Central nervous system score	2.0±1.0	2.2±0.9	2.0±1.0	0.3704
SOFA-Renal score	0.8±1.1	0.8±1.3	0.7±1.1	0.9333
SOFA-Coagulation score	0.2±0.4	0.2±0.4	0.2±0.5	0.6426
SOFA-Hepatic score	0.2±0.5	0.2±0.4	0.2±0.5	0.2953
Length of stay in ICU days	16±8	15±8	16±8	0.6151
28-day mortality (%)	15	12	17	0.1871
**Organ support-free days**				
Ventilator-free days	5±5	4±4	5±5	0.7034
Dialysis-free days	14±8	14±8	15±8	0.5937
Vasopressor-free days	12±7	11±7	12±7	0.6733
ECMO-free days	15±8	15±8	15±8	0.5753
**Inflammatory values**				
Leucocytes (1000/µl)	12±4	11±3	13±4	0.0206
CRP (mg/l)	138±90 (51)	122±94 (15)	145±89 (36)	0.9442
Procalcitonin (ng/dl)	2.5±7.0 (130)	3.2±6.9 (45)	2.1±7.0 (85)	0.5102
**Kidney values**				
Urine output (ml/day)	2882±1249	2922±1155	2863±1298	0.3192
Urine output (ml/kg/hr)	1.6±0.8	1.6±0.8	1.5±0.9	0.5337
Creatinine (mg/dl)	1.2±1.0	1.4±1.3	1.2±0.8	0.1127
**Liver values**				
AST (GOT) (IU/l)	115±210 (80)	169±354 (21)	96±125 (59)	0.2890
ALT (GPT) (IU/l)	79±125 (145)	80±178	79±87 (96)	0.9813
Bilirubin (mg/dl)	0.8±0.9	0.8±1.0	0.8±0.9	0.7605

7. Disease severity – severe ARDS (Adjusted).

Linear regression – with Age, sex, BMI, SOFA_d1, Apache, Statin, History of stroke (varied comorbidity at baseline) and rs4957796 genotype.

Variable	All	Severe ARDS		p Value
		T/C|C/C	T/T	
	(n=95)	(n=27)	(n=68)	
SOFA	8.6±3.7	7.8±2.9	8.9±3.9	0.1927
SOFA-Respiratory score	2.6±0.5	2.5±0.6	2.6±0.5	0.2433
SOFA-Cardiovascular score	1.8±1	1.7±0.9	1.9±1.1	0.6632
SOFA-Central nervous system score	2.4±1	2.1±1	2.5±0.9	0.0909
SOFA-Renal score	0.8±1	0.8±0.8	0.8±1.1	0.6701
SOFA-Coagulation score	0.4±0.6	0.2±0.4	0.5±0.7	0.0680
SOFA-Hepatic score	0.3±0.7	0.4±0.6	0.3±0.7	0.7948
Length of stay in ICU days	21±16	19±12	22±18	0.2269
28-day mortality (%)	29	11	37	0.0349
**Organ support-free days**				
Ventilator-free days	2±3	3±2	2±3	0.4343
Dialysis-free days	15±8	15±7	16±8	0.3880
Vasopressor-free days	10±7	10±5	10±7	0.3774
ECMO-free days	16±9	16±8	15±9	0.8785
**Inflammatory values**				
Leucocytes (1000/µl)	13±4	12±3	13±4	0.1942
CRP (mg/l)	164±107 (35)	113±86 (8)	179±110 (27)	0.4006
Procalcitonin (ng/dl)	4.4±10.9 (90)	3.2±7.6 (24)	4.9±11.9 (66)	0.7463
**Kidney values**				
Urine output (ml/day)	2955±1264	3388±1267	2783±1231	0.0698
Urine output (ml/kg/hr)	1.4±0.7	1.7±0.7	1.3±0.6	0.0269
Creatinine (mg/dl)	1.2±0.7	1.3±0.7	1.2±0.7	0.6870
**Liver values**				
AST (GOT) (IU/l)	426±1430 (60)	296±588 (14)	465±1605 (46)	0.7366
ALT (GPT) (IU/l)	124±243 (93)	157±329	111±199 (66)	0.4093
Bilirubin (mg/dl)	1.4±2.9	1.1±1.4	1.6±3.3	0.5769

Supplementary Figure 1

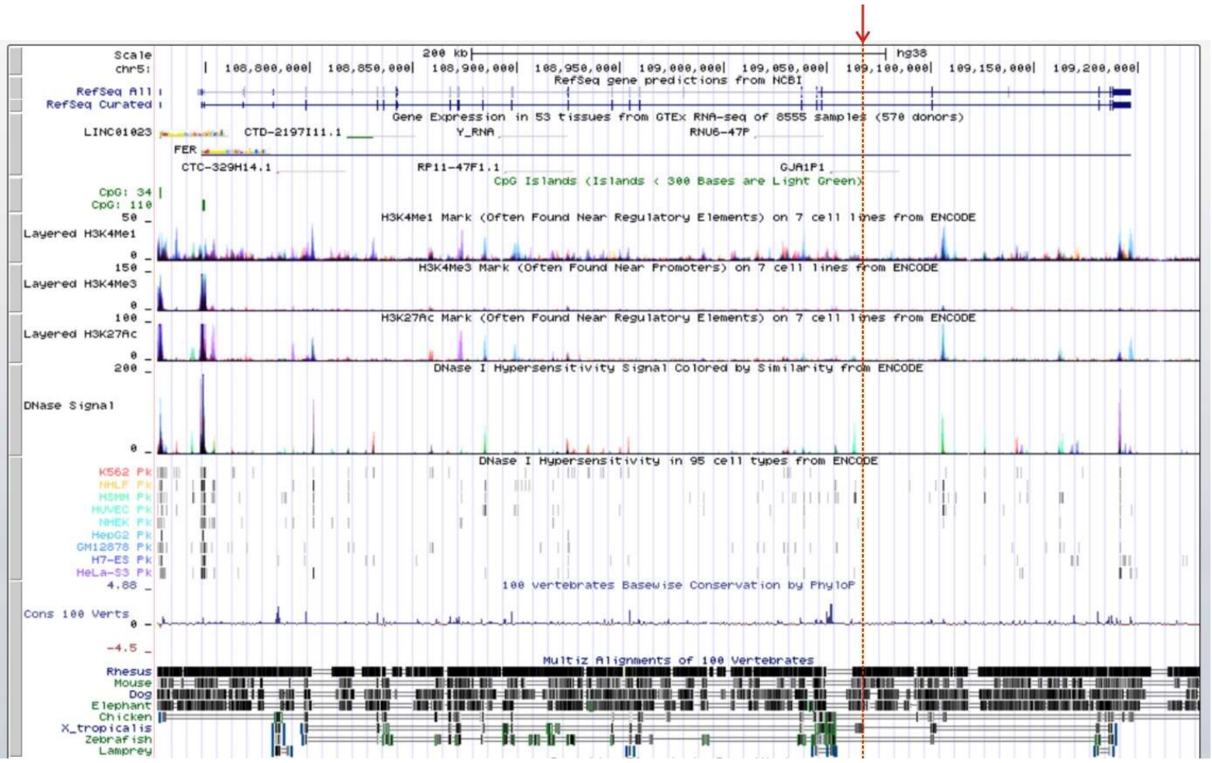

Supplementary figure 1. The figure shows the localization of the rs4957796 polymorphism (indicated with red arrow) within the FER gene and in relation to the known regions of histone modifications, DNase hypersensitivity, and interspecies conservation. The data were generated from the ENCODE Integrated Regulation track and Vertebrate Conservation track for the human genome reference sequence GRCh38/hg38; visualized using the UCSC browser (https://genome.ucsc.edu).
